# Parental education is associated with differential engagement of neural pathways during inhibitory control

**DOI:** 10.1038/s41598-021-04152-4

**Published:** 2022-01-07

**Authors:** Christopher N. Cascio, Nina Lauharatanahirun, Gwendolyn M. Lawson, Martha J. Farah, Emily B. Falk

**Affiliations:** 1 School of Journalism and Mass Communication, University of Wisconsin-Madison, 5115 Vilas Hall, 821 University Ave., Madison, WI 53706 USA; 2 Biobehavioral Health, Pennsylvania State University, 531 Chemical & Biomedical Engineering building, University Park, PA 16803 USA; 3 Children’s Hospital of Philadelphia, Perelman School of Medicine, University of Pennsylvania, 3400 Civic Center Blvd., Philadelphia, PA 19104 USA; 4 Department of Psychology, University of Pennsylvania, 3710 Hamilton Walk, Philadelphia, PA 19104 USA; 5 Annenberg School for Communication, University of Pennsylvania, 3620 Walnut Street, Philadelphia, PA 19104 USA

**Keywords:** Social neuroscience, Psychology

## Abstract

Response inhibition and socioeconomic status (SES) are critical predictors of many important outcomes, including educational attainment and health. The current study extends our understanding of SES and cognition by examining brain activity associated with response inhibition, during the key developmental period of adolescence. Adolescent males (*N* = 81), aged 16–17, completed a response inhibition task while undergoing fMRI brain imaging and reported on their parents’ education, one component of socioeconomic status. A region of interest analysis showed that parental education was associated with brain activation differences in the classic response inhibition network (right inferior frontal gyrus + subthalamic nucleus + globus pallidus) despite the absence of consistent parental education-performance effects. Further, although activity in our main regions of interest was not associated with performance differences, several regions that were associated with better inhibitory performance (ventromedial prefrontal cortex, middle frontal gyrus, middle temporal gyrus, amygdala/hippocampus) also differed in their levels of activation according to parental education. Taken together, these results suggest that individuals from households with higher versus lower parental education engage key brain regions involved in response inhibition to differing degrees, though these differences may not translate into performance differences.

## Introduction

Socioeconomic status (SES) is a critical factor in determining an individual’s access to both economic and social resources^1^. Across both epidemiological and psychological studies, early socioeconomic disadvantage is associated with health risk behaviors^2,3^, premature mortality^4,5^, poor cardiovascular health^6^, lower levels of achievement later in life^7^, depression^8^ and other long-lasting health conditions such as substance use dependence^4,5,9–11^. Socioeconomic disadvantage also shapes critical neural pathways underlying core executive functions including the ability to override prepotent responses, known as response inhibition, a type of inhibitory control^12–16^. Although previous literature has identified a relationship between SES and response inhibition^12–16^, different indicators of SES may relate to response inhibition in the brain in different ways. Thus, additional research is needed to further elucidate how individual indicators of SES relate to response inhibition processes in the brain, which is the focus of the current manuscript.

Indicators of SES typically include parental education, occupation, and household income, which are typically intercorrelated with one another^1^. In this study, we focus on parental education as it is often used as an index of early socioeconomic disadvantage and is fairly stable from childhood through adolescence^17^. High levels of parental education are associated with greater access to nonmaterial resources (e.g., experiences, skills, knowledge^18^), as well as material resources (e.g., healthcare, private schooling^19^). Higher levels of parental education are also positively related to children’s academic achievement^20^, and negatively associated with risky behaviors and impulsive decision making in adulthood^21^.

Response inhibition is a key executive function that often facilitates positive health outcomes by reducing maladaptive risky behavior. Response inhibition is the ability to resist impulsive behavioral responses in service of responses that are consistent with a person’s goals^14,15,22,23^. Response inhibition is a type of inhibitory control distinct from attentional inhibition (typically measured using interference tasks), which refers to the ability to resist interference or distractions within a person’s environment^14,15,23^. If impaired, response inhibition can lead to poor physical and mental health outcomes, such as obesity and overeating^24–26^, as well as substance use^27–29^.

At the neural level, several brain regions have been associated with successful response inhibition, including the basal ganglia, superior, middle and inferior frontal gyri, precentral gyrus, inferior parietal lobule, insula, angular gyrus, supramarginal gyrus, superior and middle temporal gyri, pre-supplementary motor area, and anterior cingulate cortex (for reviews, see^30–33^). Among these brain regions, one primary pathway, the frontal-subcortical pathway, has been consistently related to response inhibition in existing samples^30^. The frontal-subcortical pathway discussed by Aron & Poldrack (2006) includes right lateralized activity in the inferior frontal gyrus (IFG), which excites the subthalamic nucleus (STN), and in turn excites the globus pallidus (GP) during response inhibition.

Some evidence relates early socioeconomic indicators to the development of inhibitory control and its underlying mechanisms. Environmental and context effects may be particularly impactful during adolescence when prefrontal functioning is developing at a slower rate relative to subcortical brain areas involved in reward and motivation^34–37^. Children who experience early socioeconomic adversity, as indexed by low socioeconomic status, also show decreased prefrontal cortex functioning during an interference task relative to those from high socioeconomic households^38^. Another study found that adolescent females from low socioeconomic status backgrounds showed greater anterior cingulate cortex activation, which was associated with decreased response inhibition performance, suggesting that environmental conditions such as socioeconomic status play a role in shaping neurocognitive abilities^39^.

However, only a limited amount of research examines SES and response inhibition and results so far have been mixed concerning SES effects on task performance, brain activity, and the relation between the two measures^39–41^. Thus, more research is needed that explores these interrelations among these measures. Some research shows that adolescents experiencing higher levels of poverty indexed by household income, compared to those with lower levels of poverty showed a negative relationship between puberty and neural inhibitory control during a multi-source interference task^41^. This finding suggests that poverty may accelerate development leading to worse inhibitory control, specifically attentional inhibition. In terms of parental education, a recent behavioral study showed that higher parental education was related to a higher level of response inhibition in a stop signal task especially for African American adolescents relative to Caucasian adolescents^42^. In addition, research by Tominson et al. (2020) examined the relationship between parental income, education, and neighborhood poverty on bilateral IFG activation response inhibition performance and found that greater neighborhood poverty was associated with decreased activity in the IFG, and IFG activity during inhibitory control (no-go > go) was positively correlated with inhibitory performance. However, parental education was not associated with IFG activity during inhibitory control or with inhibitory performance. Other recent studies showed that poverty is associated with performance on cognitive control tasks^43^, and that lower SES is associated with reduced inhibitory control on behavioral tasks, despite not finding corresponding effects in the brain^44^. Although these results support the notion that early socioeconomic adversity of different types may shape the development of inhibitory control, more research is needed regarding how parental education affects behavioral and brain measures of response inhibition, a key executive function in avoiding risky behaviors and protecting health.

In this study, we aim to address this need by examining the effects of parental education as an index of early socioeconomic advantage or disadvantage on neurobehavioral correlates of response inhibition in a go-no-go task^45,46^. We examined the relationship between brain activity measured with fMRI during a go-no-go response inhibition task^45,46^ and participants’ reports of their parents’ education levels in a sample of male teenagers. If parental education is indicative of socioeconomic status and promotes development of regulatory control mechanisms such as response inhibition, then we would expect increased activation of the frontal-subcortical pathway underlying response inhibition in those with high, relative to low, levels of parental education. Our primary analyses examined whether parents’ education was associated with neural activity in the frontal-subcortical pathway during response inhibition. Second, we examined whether neural activity in the frontal-subcortical pathway during response inhibition varies by behavioral inhibitory performance. Third, we examined whether the relationship between neural activity in the frontal-subcortical pathway and task performance depended on parents’ education. Finally, we conducted exploratory whole brain analyses to examine additional relationships between neural correlates of response inhibition and parental education.

## Results

### Inhibitory performance

Our primary measure of inhibitory performance was efficiency during the go-no-go task, calculated as the average go trial reaction time divided by the proportion of correctly inhibited no-go trials^40,47^. Efficiency scores ranged from 0.34 to 0.73 (*M* = 0.50, *SD* = 0.08; lower indicates better performance). The correct percentage of inhibitory responses made on the go/no-go task ranged from 46.7 to 96.7% (*M* = 75.8%, *SD* = 11.5%; higher is better). The average response time on go trials ranged from 0.26 to 0.54 s (*M* = 0.38 s, *SD* = 0.04 s; lower is faster). Overall, the range of scores on our behavioral measures suggests that there were not floor or ceiling effects associated with performance.

### Parents’ education

Participants’ self-reports of their parents’ education was measured as the average score between the fathers’ and mothers’ education. Parents had an average education of a bachelor’s degree (*M* = 4.78, *SD* = 1.16), ranging from an average of a high school diploma (*min* = 2) to an average of a graduate degree (*max* = 6). Independently, fathers and mothers had an average education of a bachelor’s degree (*M* = 4.87, *SD* = 1.38; *M* = 4.69, *SD* = 1.30; respectively), both ranging from a high school diploma (*min* = 2) to a graduate degree (*max* = 6). Fathers’ and mothers’ education were significantly correlated (*t*(75) = 5.08, *p* < 0.001, *r* = 0.51, *CI* = [0.32, 0.66]).

### Inhibitory performance and parents’ education

**Figure 1:**
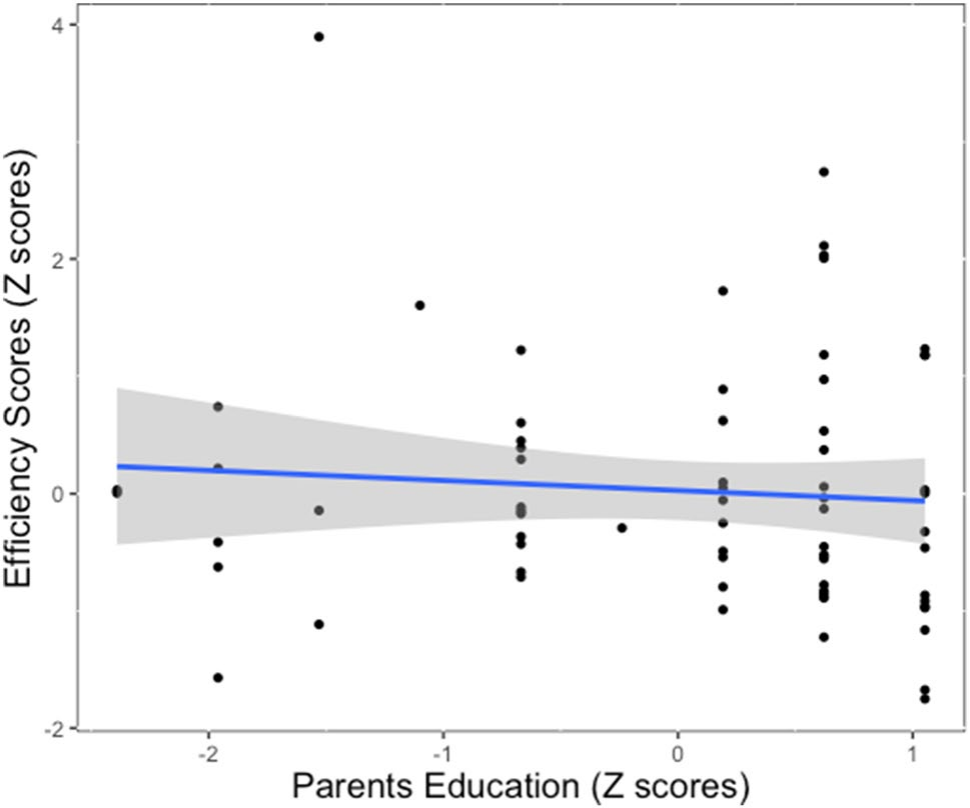
Relationship between parental education and inhibitory performance. *Note*: Scatterplot showing the relationship between parental education and individual differences in inhibitory performance (i.e., efficiency scores). Robustness checks also verified that the relationship between parental education and inhibitory performance remained nonsignificant when removing potential outliers beyond 3 SD ((*β* = − 0.00, *t*(66) = − 0.02, *p* = 0.986, *r*^*2*^ = 0.00, *CI* = [− 0.24, 0.24]), suggesting the lack of a relationship is not driven by an outlier (see scatterplot in supplemental materials). Scatterplot was created using ggplot2 in the ggplot2 (version 3.3.5) package (https://ggplot2.tidyverse.org).

We examined whether there was a relationship between performance measured during the go/no-go task and parents’ education, controlling for cohort sample. No significant relationship was found between parents’ education and our main measure of inhibitory performance, efficiency scores, (*β* = − 0.01, *t*(67) = − 0.72, *p* = 0.472, *r*^*2*^ = 0.01, *CI* = [− 0.35, 0.16]; Fig. 1). Additional analyses on sub-components of the efficiency score can be found in supplemental materials.

### ROI analyses

Our primary brain region of interest (ROI) consisted of the union of the right IFG, and sub-portions of the right BG (STN and GP), which make up the classic frontal-subcortical pathway^48^. Our primary analysis examined whether parents’ education was associated with brain activity in the right IFG, and sub-portions of the right BG (STN and GP), which make up the classic frontal-subcortical pathway^48^ during correct inhibitory trials compared to correct go trials. This analysis used the following model: ROI = β_1_(Parents’ Education) + β_2_(Study) + ε, where Study is a covariate controlling for the two cohorts pooled for these analyses.

#### Response inhibition activity and parents’ education

**Figure 2:**
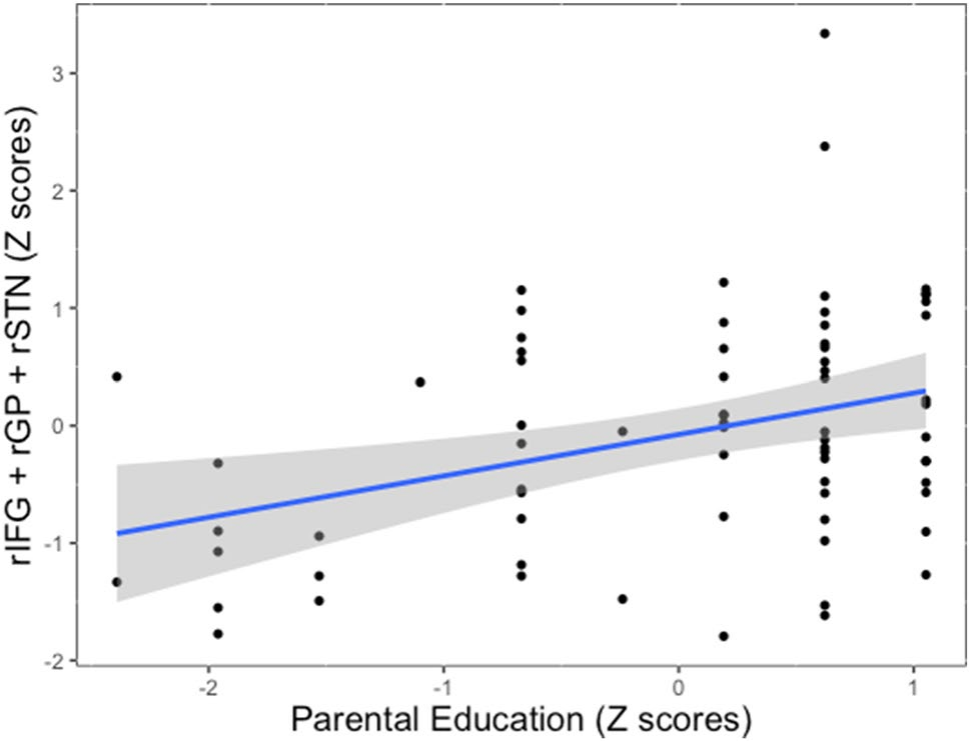
Relationship between parental education and activity in classic frontal-subcortical response inhibition pathway. *Note*: Scatterplot showing the relationship between parental education and activity in the rIFG, rSTN, and rGP during successful no-go > successful go trials. Robustness checks verified that the relationship between parental education and activity in the rIFG + rSTN + rGP remained significant when excluding potential outliers beyond 3 SD ((*β* = 0.04, *t*(67) = 3.13, *p* = 0.003, *r*^*2*^ = 0.06, *CI* = [0.12, 0.53]), suggesting the relationship is not driven by an outlier (see scatterplot in supplemental materials). Scatterplot was created using ggplot2 in the ggplot2 (version 3.3.5) package (https://ggplot2.tidyverse.org).

We replicated past findings that activity within the classic response inhibition network was greater during correct no-go (*M* = 0.088, *SD* = 0.37) versus go trials (*M* = − 0.060, *SD* = 0.52) for our sample as a whole (*F*(1, 68) = 8.17, *p* = 0.006, *η*^*2*^ = 0.03, *CI* = [0.04, 0.25]; see Supplemental Table S5 for results of a whole brain analysis of this same contrast). Next, our primary focal analysis examined whether parents’ education was associated with neural activity in the classic response inhibition regions during successful inhibitory control. Greater parental education was associated with increased activity within the response inhibition regions during correct no-go vs. go trials (*β* = 0.35, *t*(68) = 3.10, *p* = 0.003, *r*^*2*^ = 0.12, *CI* = [0.13, 0.58]; Fig. 2). Exploratory follow up analyses demonstrated that the association with parental education was primarily driven by fathers’ education (*β* = 0.44, *t*(65) = 3.88, *p* < 0.001, *r*^*2*^ = 0.19, *CI* = [0.21, 0.66]), rather than mothers’ education (*β* = 0.18, *t*(68) = 1.49, *p* = 0.140, *r*^*2*^ = 0.03, *CI* = [− 0.06, 0.43]).

#### Response inhibition activity and inhibitory performance

We next examined whether neural activity in the classic frontal-subcortical response inhibition pathway (rIFG + rSTN + rGP) during correct no-go versus correct go trials was significantly associated with behavioral performance. No significant relationship was found between the primary behavioral measure of performance (i.e., the efficiency score), and response inhibition activity in our focal regions of interest, (*β* = 0.03, *t*(67) = 0.25, *p* = 0.802, *r*^*2*^ = 0.00, *CI* = [− 0.20, 0.26]). Additional analyses on sub-components of the efficiency score can be found in supplemental materials. Whole brain analysis exploring additional regions associated with performance is described below (see “Whole brain analysis” section).

#### Response inhibition activity, parents’ education, and inhibitory performance

Finally, we examined whether the relationship between inhibitory performance and neural activity was moderated by parents’ education (Table 1). Parents’ education did not moderate the relation between inhibitory performance and brain activity in the classic inhibitory network. This analysis also confirmed that parents’ education was associated with activity within the classic response inhibition network, after controlling for inhibitory performance, and that there was not a significant relationship between inhibitory performance and neural activity when controlling for parents’ education. All regression model results were consistent when examining the three anatomical components of the response inhibition ROI separately (details can be found in supplemental materials, Tables S1–S3). Additional analyses on sub-components of the efficiency score can be found in supplemental materials.

**Table 1:** Relationships between brain activity and parents’ education.

	β	t(65)	*p*	r^2^	CI
Parents’ edu	0.36	3.09	0.003	0.13	[0.13, 0.59]
Efficiency score	0.06	0.54	0.590	0.00	[− 0.16, 0.28]
Parents’ edu X efficiency score	0.05	0.49	0.623	0.00	[− 0.16, 0.26]
Study	0.00	− 0.00	0.999	0.00	[− 0.23, 0.23]
Model: ROI = β_1_(Parents’ Edu) + β_2_(Efficiency Score) + β_3_(Parents’ Edu * Efficiency Score) + β_4_(Study) + ε					

	β	t(62)	*p*	r^2^	CI
Fathers’ edu	0.44	3.88	< 0.001	0.20	[0.21, 0.67]
Efficiency score	0.06	0.51	0.613	0.00	[− 0.16, 0.27]
Fathers’ edu X efficiency score	0.11	1.01	0.315	0.01	[− 0.11, 0.33]
Study	− 0.03	− 0.24	0.809	0.00	[− 0.26, 0.20]
Model: ROI = β_1_(Fathers’ Edu) + β_2_(Efficiency Score) + β_3_(Fathers’ Edu * Efficiency Score) + β_4_(Study) + ε					

	β	t(65)	*p*	r^*2*^	CI
Mothers’ edu	0.20	1.53	0.131	0.03	[− 0.06, 0.45]
Efficiency score	0.05	0.41	0.682	0.00	[− 0.18, 0.28]
Mothers’ edu X efficiency score	− 0.03	− 0.22	0.823	0.00	[− 0.27, 0.21]
Study	0.00	0.02	0.987	0.00	[− 0.25, 0.25]
Model: ROI = β_1_(Mothers’ Edu) + β_2_(Efficiency Score) + β_3_(Mothers’ Edu * Efficiency Score) + β_4_(Study) + ε					

ROI = our main region of interest, combining the right inferior frontal gyrus + subthalamic nucleus + globus pallidus (rIFG, rSTN, and rGP; see supplemental materials for parallel results divided into sub-regions).

Regression models examining the relationship of response inhibition activity to parents’ education, and efficiency scores, controlling for study.

In sum, those with higher parental education displayed significantly greater activity in the response inhibition regions (rIFG + rSTN + rGP) during successful inhibitory control compared to those from households with lower parental education. The relationship between parents’ education and neural activity within this pathway remained significant when controlling for individual differences in performance. Thus, although participants with higher parental education showed greater activity within the classic inhibition network during inhibitory trials, this difference was not related to performance differences. Furthermore, parents’ education did not moderate the relationship between performance and neural activity within the classic inhibition ROI.

Finally, we explored whether parents’ education and inhibitory performance (i.e., efficiency scores) was associated with different levels of recruitment of brain areas outside the classic inhibition network, and whether differential recruitment of these regions was related to performance and parents’ education, respectively. To explore these possibilities, we conducted whole brain analyses, and examined relationships between regions significantly associated with parental education and inhibitory performance.

### Whole brain analysis

#### Main effect of parents’ education on response inhibition activity

First, we examined the main effect of parents’ education on neural activity during correct no-go trials compared to correct go trials throughout the brain. Our goal was to determine whether adolescents from households with different parental education differed in neural activity beyond our hypothesized brain regions of interest. Higher parental education was associated with increased activity in the middle temporal gyrus, angular gyrus, basal ganglia, and occipital cortex (Table 2). No regions were significantly more active in those with lower parental education.

**Table 2:** Brain regions associated with higher parental education, according to the model Whole Brain Analysis = β_1_(Parents’ Edu) + β_2_(Study) + ε.

Region	Hemisphere	x	y	z	k	t
Angular gyrus	R	35	− 74	40	60	4.15
Thalamus/basal ganglia	L	− 13	− 26	1	91	4.38
Middle temporal gyrus	R	66	− 57	4	120	4.69
Occipital lobe	R	18	− 95	1	107	4.34

Whole brain analysis of parents’ education regressed onto the contrast correct no-go compared to go trials, controlling for study (*K* > 57, *p* = 0.001, corresponding to *p* < 0.05, corrected).

#### Functional ROIs and inhibitory performance

Next, we examined whether individual differences within the regions identified in Table 2 that covaried with parents’ education were associated with inhibitory performance, controlling for study sample. There was a significant relationship between activity in the angular gyrus and marginal relationship between activity in the middle temporal gyrus (MTG) and efficiency scores, such that increased activity in the angular gyrus and MTG was associated with better efficiency (*β* = − 0.24, *t*(67) = − 2.07, *r*^*2*^ = 0.06, *p* = 0.043, *CI* = [− 0.45, − 0.01]; *β* = − 0.21, *t*(67) = − 1.73, *r*^*2*^ = 0.04, *p* = 0.089, *CI* = [− 0.42, 0.03]; respectively). No other regions were associated with efficiency scores, *p* > 0.250. Results pertaining to the components of efficiency (reaction time and percent of correct no-go trials) are reported in supplemental materials, Table S4.

#### Main effect of inhibitory performance on response inhibition activity

Given that activity within the classic inhibitory control ROIs was not associated with performance, might other areas of the brain play a role in inhibition and be associated with performance? To address this possibility, we examined the main effect of inhibitory performance on neural activity by correlating brain activation that was greater during correct no-go trials versus correct go trials with inhibitory performance. Results indicated that better performance (indexed by a lower efficiency score) was associated with increased activity in the angular gyrus, MTG, superior frontal gyrus, left middle frontal gyrus, ventral medial prefrontal cortex (VMPFC), and amygdala, among other regions (Table 3). No activity was significantly more strongly associated with worse performance.

**Table 3:** Brain regions associated with better inhibitory performance, according to the model Whole Brain Analysis = β_1_(Efficiency Score) + β_2_(Study) + ε.

Region	Hemisphere	x	y	z	k	t
Superior frontal gyrus	L	− 16	36	46	255	− 5.52
Angular gyrus	R	53	− 74	31	74	− 4.06
Angular gyrus	L	− 44	− 67	37	355	− 5.4
Middle frontal gyrus	L	− 40	39	− 5	74	− 4.03
VMPFC	L/R	− 16	50	4	238	− 4.55
Middle temporal gyrus	R	73	− 33	− 8	169	− 5.75
Amygdala/hippocampus	L	− 23	− 5	− 17	1045	− 6.29
Inferior temporal gyrus	L	− 37	− 2	− 35	64	− 4.56

Whole brain analysis of efficiency scores regressed onto the contrast correct no-go compared to go trials, controlling for study (*K* > 57, *p* = 0.001, corresponding to *p* < 0.05, corrected). Negative correlations are associated with better performance. No regions were significantly associated with worse performance.

#### Functional ROIs and parents’ education

Finally, we examined whether individual differences in activity within the regions identified in Table 3 that covaried with efficiency scores were associated with parents’ education. Results indicated that greater activity in the middle frontal gyrus (*β* = 0.29, *t*(68) = − 2.52, *r*^*2*^ = 0.08, *p* = 0.014, *CI* = [0.06, 0.53]), VMPFC (*β* = 0.24, *t*(68) = 2.14, *r*^*2*^ = 0.06, *p* = 0.036, *CI* = [0.02, 0.46]), amygdala/hippocampus (*β* = 0.30, *t*(68) = 2.64, *r*^*2*^ = 0.09, *p* = 0.010, *CI* = [0.07, 0.52]), and MTG (*β* = 0.28, *t*(68) = 2.45, *r*^*2*^ = 0.08, *p* = 0.017, *CI* = [0.05, 0.51]) were significantly associated with higher parental education. Full results are reported in Table 4. Given the fROIs relationship to inhibitory performance (i.e., efficiency scores) and parents’ education, exploratory analyses were run to examine whether there was an indirect relationship between parents’ education and inhibitory performance through neural activity in these regions. Results indicated that there were significant indirect effects of parental education on inhibitory performance through activity in the middle frontal gyrus (*β* = − 0.19, *p* = 0.016, *CI* = [− 0.37, − 0.04]), VMPFC (*β* = − 0.16, *p* = 0.036, *CI* = [− 0.34, 0.01]), amygdala/hippocampus (*β* = − 0.23, *p* = 0.003, *CI* = [− 0.43, − 0.06]), and MTG (*β* = − 0.19, *p* = 0.014, *CI* = [− 0.37, − 0.04]); See supplemental materials for full results from the tests of indirect effects.

**Table 4:** Functional ROIs identified in the model (Whole Brain Analysis = β_1_(Parents’ Edu) + β_2_(Study) + ε) associated with parents’ education.

	β	t(68)	*p*	r^2^	CI
Superior frontal gyrus	0.17	1.55	0.126	0.03	[− 0.05, 0.38]
Angular gyrus	0.20	1.83	0.072	0.04	[− 0.02, 0.43]
Middle frontal gyrus	0.29	2.52	0.014	0.08	[0.06, 0.53]
VMPFC	0.24	2.14	0.036	0.06	[0.02, 0.46]
Middle temporal gyrus	0.28	2.45	0.017	0.08	[0.05, 0.51]
Amygdala/hippocampus	0.30	2.64	0.010	0.09	[0.07, 0.52]
Inferior temporal gyrus	0.14	1.13	0.263	0.02	[− 0.12, 0.43]

Relationship between functional ROIs defined by regions that were significantly associated with efficiency scores in the whole brain analysis (fROIs = β_1_(Efficiency Scores) + β_2_(Study) + ε) in Table 3 and measures of parents’ education. Parallel results were found for fathers’ education. No regions were significantly related to mothers’ education.

## Discussion

Previous research supports the idea that differences in a family’s socioeconomic resources may shape the development of children’s cognitive control and executive function more broadly^38,40–42,49^. However, limited knowledge exists regarding how different components of socioeconomic status relate to behavioral and brain measures of response inhibition^40^, a key executive function that often facilitates positive health outcomes by reducing maladaptive risky behaviors. In this study, we examined the effects of parental education as an index of early socioeconomic advantage or disadvantage, associated with access to nonmaterial resources (e.g., experiences, skills, knowledge^18^) and material resources (e.g., healthcare, private schooling^19^), on neurobehavioral correlates of response inhibition in a go-no-go task. Although we found that parental education was associated with neural activity in the frontal-subcortical response inhibition pathway (rIFG + rSTN + rGP), this did not translate into performance differences, which we discuss in more detail below.

First, we replicated prior findings showing that, on average, core regions of the classic frontal-subcortical response inhibition pathway (rIFG + rSTN + rGP) were more activated during successful no-go vs. go trials. These findings support the hypothesis that increased neural activity in the rIFG + rSTN + rGP is associated with successful inhibition and suggest that our sample in aggregate is consistent with past reports^30–33^.

Second, we tested whether participants whose parents had more or less education recruited the frontal-subcortical response inhibition pathway to differing degrees during successful inhibition. Our focal analysis showed that adolescents with more educated parents displayed significantly more activity in these classic response inhibition regions during successful inhibition. These findings are broadly consistent with previous research on SES and inhibitory control. For example, 14-year-olds at an advanced pubescent stage, who were from households with lower economic resources, showed decreased recruitment of neural regions associated with inhibitory control during a multisource-interference task. This included activity in the IFG, among other regions^41^. Research on children and adolescents aged 7–18 also found that greater neighborhood poverty (i.e., lower income) was associated with lower activation of the IFG compared to those from higher income neighborhoods, however, parental education was not associated with IFG activity^40^. The findings from the current study extend these lines of research to include parental education (which is related to access to nonmaterial^18^ and material resources^19^) as an additional factor that is associated with differences in the neural correlates of inhibitory control among adolescents.

Complementing our region of interest approach, we also conducted a whole brain analysis to determine whether neural regions outside of our hypothesized rIFG + rSTN + rGP pathway were recruited differently depending on parents’ education. Those with more educated parents displayed significantly more activity in part of the basal ganglia (i.e., putamen), among other regions, during successful response inhibition, consistent with the idea that the basal ganglia are more strongly associated with response inhibition^30,32,33^ for participants from households with higher parental education. Adolescents with more educated parents also showed increased activity in the right MTG and right angular gyrus, during successful inhibition.

Follow up analyses showed that in addition to differing in their level of activation according to parental education, increased activity in the angular gyrus was significantly associated with greater efficiency overall, and significantly faster reaction times (see supplemental materials). A similar marginal association was observed for MTG. MTG and the angular gyrus are multimodal association areas that play multiple roles in cognition. Relevant to the current findings, in past meta-analytic research examining neural correlates associated with different variants of stop signal tasks, the MTG has been implicated in successful response inhibition for go/no-go tasks that involve complex stop signal rules^32^. In addition, increased activity in the angular gyrus has been implicated in response inhibition tasks involving working memory^31^. As such, these data are consistent with our broader finding that adolescents from higher versus lower parental education backgrounds in this sample more strongly recruited brain regions implicated in response inhibition.

Further, we examined whether neural activity in the rIFG + rSTN + rGP was associated with individual differences in response inhibition performance. We expected that increased activity in the subcortical response inhibition pathway would be associated with better task efficiency. However, that was not found here. Other studies have reported a lack of such a relation as well^41,50^, raising the possibility that increased activity within the subcortical response inhibition pathway does not in fact contribute to better performance. It is nevertheless true that another study of SES, inhibitory control and brain activity did find a positive relation between inhibitory control and IFG activity^40^.

To further examine the relationship between neural processes associated with response inhibition, performance (i.e., efficiency scores), and parental education we conducted a whole brain analysis that examined the main effect of inhibitory performance on neural activity during correct no-go trials compared to correct go trials. In addition to the MTG and angular gyrus identified above, several other regions were significantly associated with better efficiency, including the superior frontal gyrus, middle and inferior temporal gyrus, middle frontal gyrus, ventral medial prefrontal cortex (VMPFC), and amygdala/hippocampus. Among these regions, follow up ROI analyses found that increased activity in the middle frontal gyrus, MTG, VMPFC, and amygdala/hippocampus were significantly associated with greater parental education. Further, despite the lack of a direct effect, exploratory analyses revealed that there was an indirect effect of parental education on inhibitory performance, through brain activity in the middle frontal gyrus, VMPFC, amygdala/hippocampus, and MTG. The middle frontal gyrus has been associated with correct inhibitory processing^31^ and increased activity in the VMPFC and amygdala/hippocampus (i.e., fronto-limbic activation) has been associated with inhibitory processing of emotional stimuli compared to neutral stimuli^51^.

Our exploratory whole brain analyses showing there was an indirect effect of parental education on inhibitory performance, through brain activity in the middle frontal gyrus, VMPFC, amygdala/hippocampus, and MTG also suggest that different brain regions might be relevant to performance for different indicators of SES. For example, although Tomlinson et al. (2020) did not find that activity in IFG was associated with parental education, they did observe differences in the IFG during correct inhibitory control based on neighborhood poverty levels, and also found that these differences were associated with behavior. In parallel, Spielberg et al. (2015) found increased activity in the anterior cingulate during response inhibition over a two-year period was associated with poorer inhibitory control among females, but not males, from lower SES backgrounds based on the Hollingshead Four-Factor Index of SES, which includes household education and occupational prestige. In addition, behavioral studies of inhibitory performance and SES (i.e., including parental education, income, and neighborhood poverty) have found significant relationships between lower SES and lower inhibitory control performance^39,42–44^.

Given previous findings that vary with respect to tasks, SES measures and even gender, the present findings could not have been anticipated. Parental education predicted brain activity in the frontal-subcortical response inhibition pathway, but this activity was not accompanied by behavioral differences in our sample of male teens. Nor were they accompanied by compensatory activity detected in other brain areas for those with lower parental education. Lower SES samples have shown faster maturity in other contexts^52^. One possibility is that if lower SES is associated with faster maturity, more mature brains may show less activation for a similar level of performance. However, future research is needed to explicitly test this idea. Of course, as already noted, activity in the frontal-subcortical response inhibition pathway may not always be the limiting factor on performance.

On the other hand, it is also possible that differences in brain activity are more sensitive measures of inhibitory processes, and we did observe that activation in regions tracking inhibitory performance also varied by SES. It is possible that in a larger sample of participants, from an even wider range of SES backgrounds, would reveal differences in performance by parental education, or that differences in performance might emerge over time. It is also possible that specific components of the inhibitory process may differ by a person’s parental education levels. It may be the case that individuals from low and high parental education backgrounds simply take different approaches to successful inhibitory control; differing approaches could result in different degrees and patterns of activation despite the absence of overall differences in efficiency. For example, functional differences could be a result of different learned cognitive strategies rather than environmental factors altering neural functioning^53^. This line of reasoning is consistent with the idea that social and economic environments shape the way we think^54^, and behave^55^.

Further, we suggest that going beyond differences in performance, to differences in what gives rise to performance, has implications for intervention. Based on our brain imaging results in this study, an intervention or educational program that works to increase inhibitory control with teens from one level of SES cannot be assumed to work at a different level, especially since we observed different patterns of activation for participants’ with high and low parental education despite similar performance.

Second, given that so much imaging research is carried out at R1 universities, where the subject populations are disproportionately from educated, middle/upper-middle class families, the brain-behavior relations inferred may not in fact be representative of the whole national population. Our findings alert us to the possibility that the literature (and meta-analyses like Neurosynth) may not be fully generalizable.

Future research that includes wider samples beyond our relatively focal sample of recently-licensed (16–17 year old), male, drivers will help address whether, and for whom, the neural differences observed translate into performance differences. In addition, though the current study focused on parental education as an index of early socioeconomic advantage or disadvantage, additional measures of SES, including parental income and occupation or neighborhood socioeconomic conditions may help further clarify the relationship between inhibitory processing in the brain and inhibitory performance. For example, it may be the case that parental education is a less robust predictor of inhibitory performance in comparison to other indices of SES, such as neighborhood poverty levels as found in Tomlinson et al. (2020). Taken together with past findings, our data highlight the value of accounting for SES when planning interventions, and testing the assumption that effects are equally effective across groups. These findings collectively highlight the importance of measuring and including participants from a wider range of SES in neuroscience investigations.

Given their relationships to both parents’ education and performance in this dataset, the MTG and angular gyrus may be key regions to focus on in future studies that aim to understand relationships between parental education, brain activity and performance. In addition, adolescence is an important developmental stage where prefrontal brain areas are not fully developed^35,37^. Future studies should build upon the present study’s findings to examine whether socioeconomic factors such as parents’ education may affect key brain systems implicated in health risk behaviors.

Interestingly, the current data found that functional differences in response inhibition processing were associated with parental education, mainly driven by fathers’ rather than mothers’ education. Although parental education is one of the main indices of SES^56^, mothers’ education is often found to be associated with SES-related health differences^57^. It is possible that fathers’ education mapped more closely to these adolescents’ SES environments, given that this sample contained only male participants. For example, it may be that sons’ connectedness to their fathers could be driving these effects. Research on gender socialization suggests that parents tend to have a greater influence on same-sex children^58^ and educational attainment of mothers is less influential on their son’s educational attainment compared to daughter’s^59^. However, the relationship between parental education and neural functioning should be more closely examined in future studies.

## Conclusion

Results from the current study extend recent findings that have begun to examine the relationship between socioeconomic advantage and disadvantage and the brain. We found that although behavioral efficiency did not differ, adolescents from households with lower parental education displayed significantly less activity in classic response inhibition regions (rIFG + rSTN + rGP) during a go/no-go task compared to adolescents from households with higher parental education. Furthermore, when examining whether neural activity associated with parents’ education during correct no-go trials compared to go trials was associated with inhibitory performance, we found that increased neural activity in the MTG and angular gyrus were associated with marginally better efficiency, and significantly faster reaction times. Finally, we conducted an exploratory whole brain analysis that examined neural activity associated with inhibitory performance (i.e., efficiency scores) during correct no-go trials compared to go trials. We found that parents’ education had indirect effects on inhibitory performance through increased activity in the VMPFC, middle frontal gyrus, middle temporal gyrus, and amygdala/hippocampus. Taken together, these results show that individuals from higher and lower SES backgrounds recruit inhibitory control regions in the brain to differing degrees. Given that most studies of response inhibition have not included measures of SES, and that many studies of inhibitory performance have been conducted at R1 universities with relatively higher SES samples, there is a need for future studies to further examine the role of SES on neural processes supporting successful response inhibition, including parsing different components of SES such as parental education, income and other factors^1^.

## Methods

### Participants

Adolescent males (*N* = 81) between the ages of 16 to 17 years old across two cohorts were recruited through the University of Michigan Transportation Research Institute as part of a series of larger studies examining teen driving behavior^60–64^. All participants were right-handed, did not suffer from claustrophobia, were not currently taking any psychoactive medications, had normal (or corrected to normal) vision, did not have metal in their body that was contraindicated for fMRI, and did not typically experience motion sickness, which could affect driving simulation testing (which is not the focus of the current manuscript; these data were collected as part of a larger study about adolescent risk^60,64^). Of the original 81 participants recruited across two cohorts, 10 participants did not have useable fMRI data (6 due to technical issues with the task, 3 due to technical issues with the scanner, and 1 due to excess head motion). Removing these participants resulted in a final sample size of n = 71. Legal guardians provided written informed consent and teens provided written assent.

### Procedures

#### Study design

After participants gave assent to participate in the study, and parents provided written consent, they completed an initial fMRI scanning session appointment. At this appointment, they completed a number of online surveys prior to the fMRI scan that examined attitudes and behaviors related to driving and peer influence that are not the focus of the current manuscript. Next, they completed a response inhibition task (go/no-go) in an fMRI scanner. Finally, they completed additional post-scan online survey measures including questions regarding parents’ education. All study procedures were approved by the University of Michigan IRB and performed in accordance with relevant guidelines and regulations.

#### Parents’ education

Parents’ education served as our primary indicator of socioeconomic advantage or disadvantage. Participants were asked what level of education their father and mother each had completed on separate 7-point scales, where 1 = less than high school, 2 = high school, 3 = trade school, 4 = associates degree, 5 = bachelor’s degree, 6 = graduate degree, and 7 = unknown. A combined parents’ education variable was created using the average score between the father and mother. Unknown levels of education (response = 7) were not included in the combined parents’ education score. All participants reported parental education for at least one parent.

#### Response inhibition task

**Figure 3:**
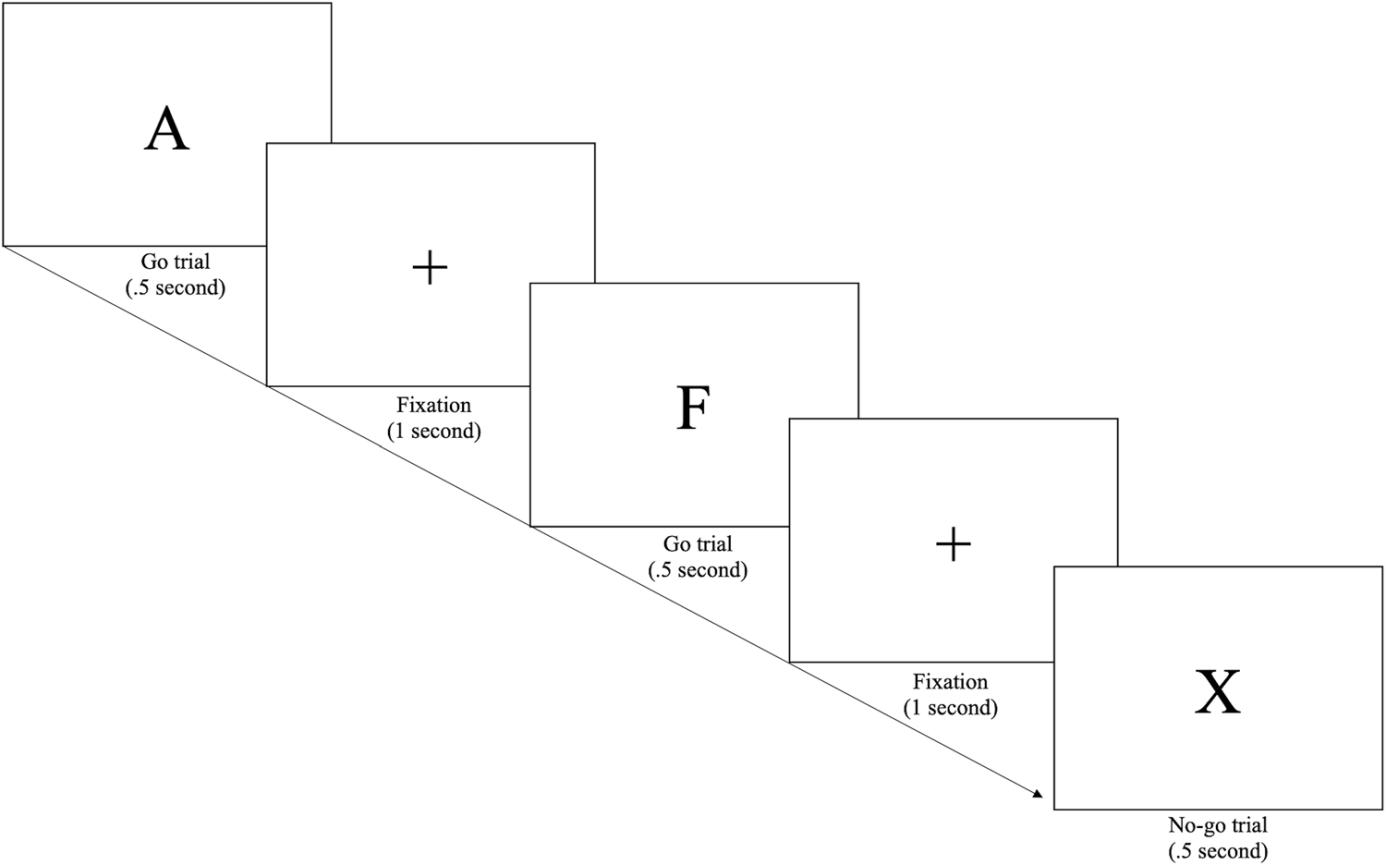
Response inhibition task. *Note*: Response inhibition was measured using the go/no-go task. We focus on the contrast (correct no-go trials > correct go trials).

During the fMRI scan participants completed the go/no-go response inhibition task^45,46^. Participants were fitted with a scanner-compatible 5-finger response glove to record behavioral responses. On each trial (464 total trials per participant), participants were presented with one alphabetic character at the center of the display. On go trials (letters A through F), participants were instructed to respond by pressing a button on the response box with their right index finger. On no-go trials (letter X), participants were instructed not to make any response (Fig. 3). Eighty percent of trials were go trials to build the habit of button pressing; the remaining 20% were no-go trials to test response inhibition. The first and last twelve trials of each block were always go trials. Each letter was presented for 0.50 s, followed by a fixed inter-stimulus interval of 1.00 s. Participants were instructed to respond to each trial before the beginning of the subsequent trial. Each no-go trial was separated from the next no-go trial by three to seven intervening go trials. Conditions of interest were correct no-go trials, false-alarm no-go trials, and miss go trials.

#### Behavioral outcomes

Inhibitory performance on the go/no-go task was measured by combining reaction time during go trials with the proportion of correctly inhibited no-go trials compared to the total number of no-go trials to create “efficiency scores” (average go trial reaction time divided by the proportion of correctly inhibited no-go trials), which takes into consideration the tradeoff between speed and accuracy^40,47^. Lower scores indicate greater efficiency (i.e., better performance).

#### fMRI data acquisition

Imaging data were acquired using a 3 Tesla GE Signa MRI scanner. Two functional runs were acquired for each participant (174 volumes per run). Functional images were recorded using a reverse spiral sequence (TR = 2000 ms, TE = 30 ms, flip angle = 90°, 43 axial slices, FOV = 220 mm, slice thickness = 3 mm; voxel size = 3.44 × 3.44 × 3.0 mm). We also acquired in-plane T1-weighted images (43 slices; slice thickness = 3 mm; voxel size = 0.86 × 0.86 × 3.0 mm) and high-resolution T1-weighted images (SPGR; 124 slices; slice thickness = 1.02 × 1.02 × 1.2 mm) for use in coregistration and normalization.

#### fMRI data analysis

To allow for the stabilization of the BOLD signal, the first four volumes (eight seconds) of each run were discarded prior to analysis. Functional images were despiked using the 3dDespike program as implemented in the AFNI toolbox. Next, data were corrected for differences in the time of slice acquisition using sinc interpolation; the first slice served as the reference slice. Data were then spatially realigned to the first functional image. We then co-registered the functional and structural images using a two-stage procedure. First, in-plane T1 images were registered to the mean functional image. Next, high-resolution T1 images were registered to the in-plane image. After coregistration, high-resolution structural images were skull-stripped using the VBM8 toolbox for SPM8 (http://dbm.neuro.uni-jena.de/vbm), and then normalized to the skull-stripped MNI template provided by FSL (“MNI152_T1_1mm_brain.nii”). Finally, functional images were smoothed using a Gaussian kernel (8 mm FWHM).

Data were modeled using the general linear model as implemented in SPM8. We modeled four trial types for each participant: correct no-go trials, false-alarm no-go trials, missed go trials, and correct go trials. The six rigid-body translation and rotation parameters derived from spatial realignment were also included as nuisance regressors. Data were high-pass filtered with a cutoff of 128 s. Random effects models averaged relevant contrasts (i.e., correct no-go trials > correct go trials) across individuals.

#### Regions of interest (ROI) analyses

**Figure 4:**
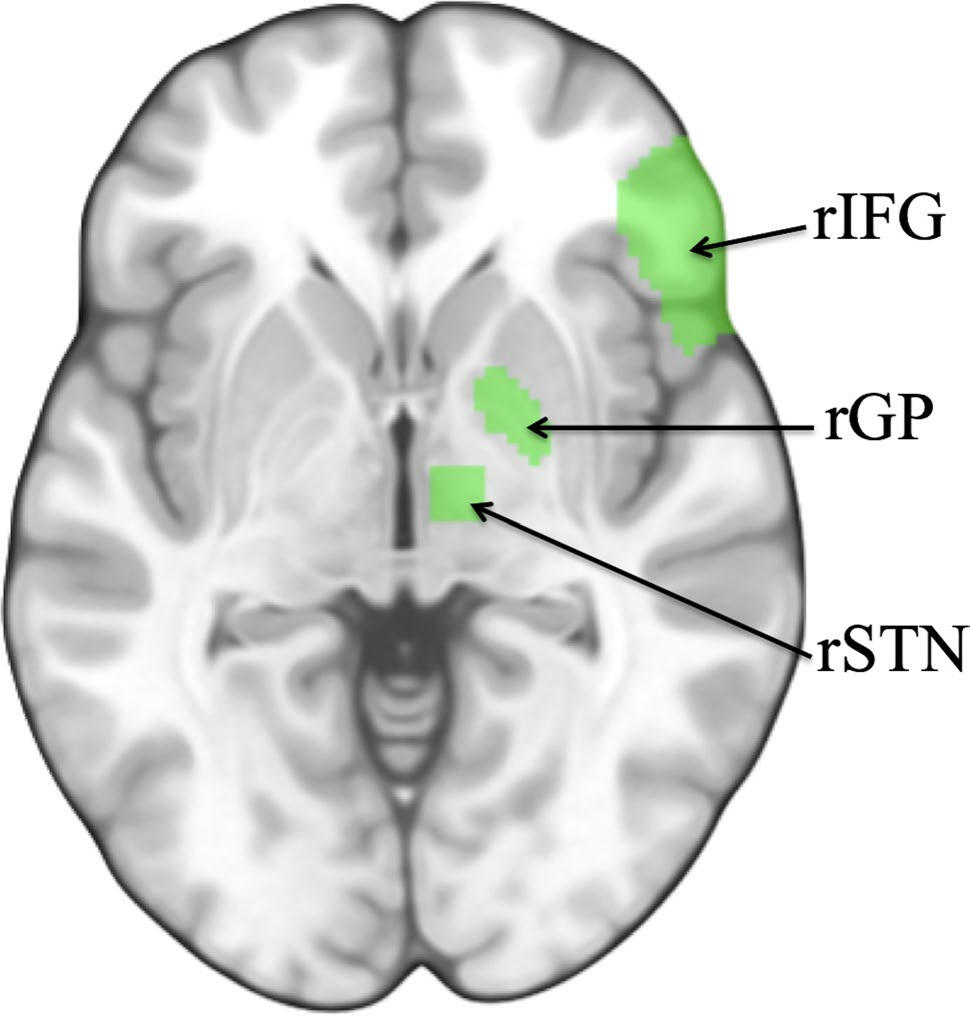
Regions of interest. *Note*: Our primary regions of interest included the right inferior frontal gyrus (rIFG), right subthalamic nucleus (rSTN), and right globus pallidus (rGP), which comprises the classic “frontal-subcortical pathway” to response inhibition^48^. ROI image was created using MRIcroGL (version 17; https://ggplot2.tidyverse.org).

Our primary region of interest (ROI) consisted of the union of the right IFG, and sub-portions of the right BG (STN and GP), which make up the classic frontal-subcortical pathway (Fig. 4)^48^. More detailed ROI definitions can be found in supplemental materials. Percent signal change (for correct no-go > correct go) was extracted from this primary ROI for each participant using MarsBaR^65^; additional analyses broken out by sub-region can be found in supplemental materials. Our primary analysis examined whether parents’ education was associated with brain activity in this a priori defined ROI during correct inhibitory trials compared to correct go trials, using the equation ROI = β_1_(Parents’ Education) + β_2_(Study) + ε, where Study is a covariate controlling for the two cohorts pooled for these analyses.

Secondary analyses examined whether activity within our main regions of interest was related to inhibitory task performance (ROI = β_1_(IP) + β_3_(Study) + ε, where IP is inhibitory performance), and whether the relationship between brain activity and task performance depended on parents’ education (ROI = β_1_(IP) + β_2_(Parents’ Education) + β_3_(Parents’ Education * IP) + β_4_(Study) + ε). All analyses were conducted in R (version 4.2.0). Linear models were fit using lm, repeated measures ANOVA was calculated using aov, and confidence intervals were calculated using confint in the Stats (version 4.2.0) package^66,67^. Beta coefficients were calculated using lm.beta in the QuantPsych (version 1.5) package^68^. Scatterplots were created using ggplot2 in the ggplot2 (version 3.3.5) package^69^. Mediation analyses were analyzed using mediate in the mediation (version 4.5.0) package^70^.

#### Whole brain analysis

In addition to our primary ROI analyses, exploratory whole brain analyses examined whether additional neural regions outside of classically reported inhibitory control regions of interest were associated with parents’ education and inhibitory performance (i.e., efficiency scores) for correct no-go trials > correct go trials (i.e., the same contrast as the ROI analyses). Accordingly, we fit a regression model predicting activation in each voxel in whole brain analyses (WBA) from parental education: WBA = β_1_(Parents’ Education) + β_2_(Study) + ε and independently from inhibitory performance: WBA = β_1_(Efficiency Scores) + β_2_(Study) + ε. The whole brain analyses are reported with a threshold of *p* = 0.001, *K* > 57, based on a Monte Carlo simulation using 3dClustSim (July 19, 2016 version), corresponding to *p* < 0.05, corrected.

In order to determine whether functional regions identified in our whole brin analyses (WBA = β_1_(Parents’ Education) + β_2_(Study) + ε; WBA = β_1_(Efficiency Scores) + β_2_(Study) + ε) were associated with efficiency scores and parents’ education, respectively, we conducted a follow up ROI analyses. Functional regions were identified based on peak clusters in Tables 2 and 3 and converted to ROI masks using the xjView toolbox (http://www.alivelearn.net/xjview). The following regression models were used to examine whether functional regions associated with parents’ education during correct no-go trials compared to go-trials were related to efficiency scores (fROI_(Parents’ Education)_ = β_1_(IP) + β_3_(Study) + ε) and whether functional regions associated with efficiency scores during correct no-go trials compared to go-trials were related to parents’ education (fROI_(Efficiency Scores)_ = β_1_(Parents’ Education) + β_3_(Study) + ε), controlling for study. Finally, exploratory mediation analyses were conducted for significant findings from our fROI analyses (fROI_(Efficiency Scores)_ = β_1_(Parents’ Education) + β_3_(Study) + ε) in order to determine whether there was an indirect relationship between parents’ education and inhibitory performance through neural activity in these functional regions. A causal mediation analysis (Monte Carlo simulations = 5,000) was conducted to examine the indirect effect (c = c’ + ab). Results for the direct effect (c’), total effect (c), and proportion mediated.

## Supplementary Information

Supplementary Information.
