# Supplementary material for: Parental education is associated with differential engagement of neural pathways during inhibitory control
Source: Sci Rep. 2022 Jan 7;12:260. doi: 10.1038/s41598-021-04152-4 (PMC8741989; doi:10.1038/s41598-021-04152-4)
Supplement: Supplementary file 1 — Supplementary Information. [file 41598_2021_4152_MOESM1_ESM.docx]

**Parental education is associated with differential engagement of neural pathways during inhibitory control**

^1^Christopher N. Cascio, ^2^Nina Lauharatanahirun, ^3^Gwendolyn M. Lawson, ^3^Martha J. Farah, & ^3^Emily B. Falk

^1^University of Wisconsin-Madison, ^2^Pennsylvania State University, ^3^University of Pennsylvania

**Supplemental Materials**

**Methods**

**Secondary inhibitory performance measures.** Secondary go/no-go performance measures consisted of reaction time (response time in milliseconds on a go trial) and percent of correct no-go trials (correct no-go trials ÷ total no-go trials).

**Regions of Interest (ROI) definitions.** The ROI stop network was defined based on past research focused on the frontal-subcortical pathway, including the rSTN (10x10x10mm box centered at MNI coordinates 10, -15, -5), rGP, and rIFG (pars opercularis and pars triangularis) ^1^. All ROIs were constructed in Wake Forest University Pickatlas toolbox within SPM ^2^. ROIs combined definitions from the Automated Anatomical Labeling Atlas (AAL) ^3^.

**Results**

**Reaction time and percent correct as secondary measures of inhibitory performance, and parental education**

We examined whether there was a relationship between secondary performance measures during the go/no-go task and parental education, controlling for study. No significant relationship was found between parental education and the percent of correct no-go trials, *p*>.250. The relationship between parents’ education and reaction time was marginal (*β*=-.21, *t*(67)=-1.77, *p*=.081, *r^2^*=.07, *CI*=[-.47, .03]), with adolescents from more educated families responding more quickly. An exploratory analysis breaking down effects by mothers’ and fathers’ education showed that although neither was associated with efficiency or errors, higher fathers’ education was associated with faster reaction times on go trials (*β*=-.24, *t*(64)=-2.03, *p*=.046, *r^2^*=.09, *CI*=[-.49, -.00]). Mothers’ education was not associated with reaction times on go trials, *p*>.250.

**ROI analyses**

**Neural response inhibition activity and inhibitory performance (secondary measures).** Next, we examined whether secondary inhibitory performance measures were associated with neural activity in the classic frontal-subcortical response inhibition pathway (rIFG+rSTN+rGP) during correct no-go trial versus correct go trials. Response inhibition ROI activity was marginally associated with the percent of correct no-go trials (*β*=-.22, *t*(67)=-1.75, *p*=.085, *r^2^*=.04, *CI*=[-.44, .03]). ROI activity was not significantly related to go response times, *β*=-.20, *t*(67)=-1.61, *p*=.112, *r^2^*=.04, *CI*=[-.41, .04]).

**Response inhibition activity (independent regions), parental education and inhibitory performance (all measures).** Next, to determine whether specific sub-regions were driving the overall effect of parental education on activity within the response inhibition network, we examined models linking parental education separately to each region in the response inhibition network (rSTN, rGP, and rIFG; tables S1, S2, S3, respectively).

Table S1. Association between parents’ education, inhibitory performance, and activity in rSTN.

|  | **β** | **t(65)** | **p** | **r^2^** | **CI** |
| --- | --- | --- | --- | --- | --- |
| Parents’ Edu | 0.25 | 2.07 | 0.043 | 0.06 | [.01, .48] |
| Efficiency Score | -0.12 | -0.97 | >0.250 | 0.01 | [-.33, .11] |
| Parents’ Edu X Efficiency Score | 0.11 | 1.02 | >0.250 | 0.01 | [-.10, .32] |
| Study | -0.05 | -0.46 | >0.250 | 0.00 | [-.29, .18] |
| Model: rSTN = β_1_(Parents’ Edu) + β_2_(Efficiency Score) + β_3_(Parents’ Edu * Efficiency Score) + β_3_(Study) + ε | | | | | |
|  | **β** | **t(65)** | **p** | **r^2^** | **CI** |
| Parents’ Edu | 0.25 | 2.14 | 0.036 | 0.06 | [.01, .49] |
| % Correct No-Go Trials | -0.05 | -0.44 | >0.250 | 0.00 | [-.28, .18] |
| Parents’ Edu X % Correct No-Go Trials | -0.13 | -1.10 | >0.250 | 0.02 | [-.37, .11] |
| Study | -0.08 | -0.68 | >0.250 | 0.01 | [-.32, .16] |
| Model: rSTN = β_1_(Parents’ Edu) + β_2_(% Correct No-Go Trials) + β_3_(Parents’ Edu * % Correct No-Go Trials) + β_3_(Study) + ε | | | | | |
|  | **β** | **t(65)** | **p** | **r*^2^*** | **CI** |
| Parents’ Edu | 0.21 | 1.72 | 0.090 | 0.04 | [-.03, .44] |
| Reaction Time | -0.23 | -1.80 | 0.077 | 0.04 | [-.44, .02] |
| Parents’ Edu X Reaction Time | 0.02 | 0.16 | >0.250 | 0.00 | [-.24, .28] |
| Study | -0.04 | -0.29 | >0.250 | 0.00 | [-.27, .20] |
| Model: rSTN = β_1_(Parents’ Edu) + β_2_(Reaction Time) + β_3_(Parents’ Edu * Reaction Time) + β_3_(Study) + ε | | | | | |

Note: Regression models examining the relationship of rSTN activity to parents’ education, and measures of inhibitory performance, controlling for study.

Table S2. Association between parents’ education, inhibitory performance, and activity in rGP.

|  | **β** | **t(65)** | **p** | **r^2^** | **CI** |
| --- | --- | --- | --- | --- | --- |
| Parents’ Edu | 0.39 | 3.45 | 0.001 | 0.15 | [.17, .63] |
| Efficiency Score | -0.04 | -0.33 | >0.250 | 0.00 | [-.25, .18] |
| Parents’ Edu X Efficiency Score | 0.07 | 0.65 | >0.250 | 0.01 | [-.14, .27] |
| Study | -0.03 | -0.23 | >0.250 | 0.00 | [-.26, .20] |
| Model: rGP = β_1_(Parents’ Edu) + β_2_(Efficiency Score) + β_3_(Parents’ Edu * Efficiency Score) + β_3_(Study) + ε | | | | | |
|  | **β** | **t(65)** | **p** | **r^2^** | **CI** |
| Parents’ Edu | 0.40 | 3.49 | 0.001 | 0.16 | [.17, .63] |
| % Correct No-Go Trials | -0.08 | -0.65 | >0.250 | 0.01 | [-.30, .15] |
| Parents’ Edu X % Correct No-Go Trials | -0.09 | -0.79 | >0.250 | 0.01 | [-.32, .14] |
| Study | -0.05 | -0.42 | >0.250 | 0.00 | [-.28, .18] |
| Model: rGP = β_1_(Parents’ Edu) + β_2_(% Correct No-Go Trials) + β_3_(Parents’ Edu * % Correct No-Go Trials) + β_3_(Study) + ε | | | | | |
|  | **β** | **t(65)** | **p** | **r*^2^*** | **CI** |
| Parents’ Edu | 0.37 | 3.22 | 0.002 | 0.13 | [.14, .61] |
| Reaction Time | -0.10 | -0.85 | >0.250 | 0.01 | [-.33, .13] |
| Parents’ Edu X Reaction Time | 0.01 | 0.11 | >0.250 | 0.00 | [-.24, .27] |
| Study | -0.02 | -0.15 | >0.250 | 0.00 | [-.25, .22] |
| Model: rGP = β_1_(Parents’ Edu) + β_2_(Reaction Time) + β_3_(Parents’ Edu * Reaction Time) + β_3_(Study) + ε | | | | | |

Note: Regression models examining the relationship of rGP activity to parents’ education, and measures of inhibitory performance, controlling for study.

Table S3. Association between parents’ education, inhibitory performance, and activity in rIFG.

|  | **β** | **t(65)** | **p** | **r^2^** | **CI** |
| --- | --- | --- | --- | --- | --- |
| Parents’ Edu | 0.30 | 2.65 | 0.010 | 0.09 | [.08, .54] |
| Efficiency Score | 0.08 | 0.71 | >0.250 | 0.01 | [-.14, .30] |
| Parents’ Edu X Efficiency Score | 0.08 | 0.69 | >0.250 | 0.01 | [-.14, .28] |
| Study | -0.25 | -2.17 | 0.034 | 0.06 | [-.48, -.02] |
| Model: rIFG = β_1_(Parents’ Edu) + β_2_(Efficiency Score) + β_3_(Parents’ Edu * Efficiency Score) + β_3_(Study) + ε | | | | | |
|  | **β** | **t(65)** | **p** | **r^2^** | **CI** |
| Parents’ Edu | 0.29 | 2.59 | 0.012 | 0.08 | [.07, .52] |
| % Correct No-Go Trials | -0.20 | -1.76 | 0.083 | 0.04 | [-.42, .03] |
| Parents’ Edu X % Correct No-Go Trials | -0.09 | -0.83 | >0.250 | 0.01 | [-.33, .13] |
| Study | -0.29 | -2.52 | 0.014 | 0.08 | [-.52, -.06] |
| Model: rIFG = β_1_(Parents’ Edu) + β_2_(% Correct No-Go Trials) + β_3_(Parents’ Edu * % Correct No-Go Trials) + β_3_(Study) + ε | | | | | |
|  | **β** | **t(65)** | **p** | **r*^2^*** | **CI** |
| Parents’ Edu | 0.27 | 2.32 | 0.023 | 0.07 | [.04, .51] |
| Reaction Time | -0.11 | -0.87 | >0.250 | 0.01 | [-.33, .13] |
| Parents’ Edu X Reaction Time | -0.01 | -0.10 | >0.250 | 0.00 | [-.27, .25] |
| Study | -0.23 | -1.95 | 0.055 | 0.05 | [-.47 .01] |
| Model: rIFG = β_1_(Parents’ Edu) + β_2_(Reaction Time) + β_3_(Parents’ Edu * Reaction Time) + β_3_(Study) + ε | | | | | |

Note: Regression models examining the relationship of rIFG activity to parents’ education, and inhibitory performance, controlling for study.

**Functional ROIs and inhibitory performance (% correct and reaction time).** We examined whether individual differences in activity within the regions identified in table 2 of the main manuscript that covaried with parental education were associated with secondary inhibitory performance measures. No significant relationships were associated with percent of correct no-go trials, *p*>.250, however greater activity in these functionally defined regions of interest were associated with faster reaction times, as reported in table S4.

**Functional ROIs indirect effect of parental education on inhibitory performance**. Exploratory analyses were run to examine whether there was an indirect relationship between parents’ education and inhibitory performance through neural activity in these regions. Results indicated that there were not significant direct effects, total effects, or proportion mediated of parental education on inhibitory performance through activity in the middle frontal gyrus (*β*=.11, *p*=.429, *CI*=[-.16, .37]; *β*=-.08, *p*=.484, *CI*=[-.31, .15]; *β*=1.15, *p*=.490, *CI*=[-19.57, 19.61], respectively), VMPFC (*β*=.09, *p*=.532, *CI*=[-.18, .35]; *β*=-.08, *p*=.498, *CI*=[-.30, .15]; *β*=.96, *p*=.514, *CI*=[-17.99, 17.20], respectively), amygdala/hippocampus (*β*=.15, *p*=.294, *CI*=[-.12, .41]; *β*=-.08, *p*=.472, *CI*=[-.31, .15]; *β*=1.41, *p*=.475, *CI*=[-19.91, 25.74], respectively), and MTG (*β*=.11, *p*=.426, *CI*=[-.16, .38]; *β*=-.08, *p*=.467, *CI*=[-.31, .15]; *β*=1.16, *p*=.476, *CI*=[-17.97, 21.73], respectively).

Table S4. Correlations between functional ROIs defined by regions that were significantly associated with parents’ education in the whole brain analysis (fROIs = β_1_(Parents’ Edu) + β_2_(Study) + ε) in Table 2 and sub-components of inhibitory performance, controlling for study.

| **Correlations with percent correct no-go trials** | | | | | |
| --- | --- | --- | --- | --- | --- |
| **Region** | **β** | **t(67)** | **p** | **r^2^** | **CI** |
| Angular gyrus | 0.01 | 0.06 | 0.953 | 0.00 | [-.23, .24] |
| Basal ganglia | -0.08 | -0.67 | 0.503 | 0.01 | [-.32, .16] |
| Middle temporal gyrus | 0.02 | 0.13 | 0.897 | 0.00 | [-.23, .26] |
| Occipital lobe | -0.10 | -0.81 | 0.419 | 0.01 | [-.34, .14] |
| **Correlations with reaction time** | | | | | |
| Angular gyrus | -0.39 | -3.51 | 0.001 | 0.15 | [-.58, -.16] |
| Basal ganglia | -0.21 | -1.75 | 0.086 | 0.04 | [-.43, .03] |
| Middle temporal gyrus | -0.31 | -2.61 | 0.011 | 0.09 | [-.51, -.07] |
| Occipital lobe | -0.24 | -1.97 | 0.054 | 0.05 | [-.45, .00] |

Note: Correlations examining the relationship between sub-components of efficiency (reaction time, correct no-go trials) and functional activity associated with parents’ education regressed onto the contrast correct no-go compared to go trials, controlling for study.

**Whole brain analyses**

**Whole brain analysis for successful no-go compared to correct go trials.** We examined the contrast (correct no-go trials > correct go trials) during the go/no-go across all participants in order to determine what the overall pattern of activity was during correct inhibitory control, correcting for multiple comparisons (*p*=.001, *K*>57). Similar to past studies of response inhibition we found significant activation in our *a priori* response inhibition regions, including the BG and rIFG. In addition, significant activation was found in the insula, ventrolateral prefrontal cortex (VLPFC), middle temporal gyrus, and inferior parietal lobe. For a full list of activations see, table S5.

Table S5. Whole brain analysis examining the contrast (correct no-go trials > correct go trials).

| **Region** | **Hemisphere** | **x** | **y** | **z** | **k** | **t** |
| --- | --- | --- | --- | --- | --- | --- |
| Middle temporal gyrus | R | 63 | -33 | -8 | 6039 | 8.97 |
| Superior temporal gyrus | R |  |  |  |  |  |
| Inferior parietal lobe | R |  |  |  |  |  |
| Inferior frontal gyrus | R |  |  |  |  |  |
| Insula | R |  |  |  |  |  |
| Basal ganglia | R |  |  |  |  |  |
| VLPFC | R |  |  |  |  |  |
| Occipital Lobe | R/L |  |  |  |  |  |
| Cerebellum | R | 35 | -43 | -35 | 243 | 5.70 |
| Middle temporal gyrus | L | -54 | -33 | -5 | 2600 | 9.07 |
| Superior temporal gyrus | L |  |  |  |  |  |
| Inferior parietal lobe | L |  |  |  |  |  |
| Inferior frontal gyrus | L |  |  |  |  |  |
| Insula | L |  |  |  |  |  |
| Basal ganglia | L |  |  |  |  |  |
| VLPFC | L |  |  |  |  |  |
| Inferior frontal gyrus | L | -37 | 8 | 25 | 80 | -6.02 |
| Precuneus | L | -23 | -67 | 37 | 256 | -5.60 |

Note: Whole brain analysis examining the contrast (correct no-go trials > correct go trials) during the go/no-go task (*K*>57, *p*=.001, corresponding to *p*<.05, corrected).

**Figures**

Figure S1. Relationship between parental education and inhibitory performance with outliers removed.

Note: Scatterplot showing the relationship between parental education and individual differences in inhibitory performance (i.e., efficiency scores). Robustness checks also verified that the relationship between parental education and inhibitory performance remained nonsignificant when removing potential outliers beyond 3 SD ((*β*=-.00, *t*(66)=-0.02, *p*=.986, *r^2^*=.00, *CI*=[-.24, .24]), suggesting the lack of a relationship is not driven by an outlier. Scatterplot was created using ggplot2 in the ggplot2 (version 3.3.5) package (<https://ggplot2.tidyverse.org>).

Figure S2. Relationship between parental education and activity in classic frontal-subcortical response inhibition pathway with outliers removed.

Note: Scatterplot showing the relationship between parental education and activity in the rIFG, rSTN, and rGP during the contrast (successful no-go > successful go trials). Robustness checks verified that the relationship between parental education and activity in the rIFG+rSTN+rGP remained significant when excluding potential outliers beyond 3 SD ((*β*=.04, *t*(67)=3.13, *p*=.003, *r^2^*=.06, *CI*=[.12, .53]), suggesting the relationship is not driven by an outlier. Scatterplot was created using ggplot2 in the ggplot2 (version 3.3.5) package (https://ggplot2.tidyverse.org).

**References**

1. Aron, A. R. & Poldrack, R. A. Cortical and subcortical contributions to stop signal response inhibition: role of the subthalamic nucleus. *J. Neurosci.* **26**, 2424–2433 (2006).

2. Maldjian, J. A., Laurienti, P. J., Kraft, R. A. & Burdette, J. H. An automated method for neuroanatomic and cytoarchitectonic atlas-based interrogation of fMRI data sets. *Neuroimage* **19**, 1233–1239 (2003).

3. Tzourio-Mazoyer, N. *et al.* Automated anatomical labeling of activations in SPM using a macroscopic anatomical parcellation of the MNI MRI single-subject brain. *Neuroimage* **15**, 273–289 (2002).
